# The Synergistic Antimicrobial Effect and Mechanism of Nisin and Oxacillin against Methicillin-Resistant *Staphylococcus aureus*

**DOI:** 10.3390/ijms24076697

**Published:** 2023-04-03

**Authors:** Jun Wang, Xinxin Ma, Jing Li, Lu Shi, Lijuan Liu, Xinyao Hou, Sijin Jiang, Pu Li, Jia Lv, Lei Han, Yue Cheng, Bei Han

**Affiliations:** 1 School of Public Health, Health Science Center, Xi’an Jiaotong University, Xi’an 710061, China; 2 Tongchuan Center for Disease Control and Prevention, Tongchuan 727031, China; 3 School of Basic Medical Sciences, Xi’an Jiaotong University, Xi’an 710061, China

**Keywords:** methicillin resistant *Staphylococcus aureus*, oxacillin, nisin, synergistic antimicrobial effect, in vitro, in vivo

## Abstract

Methicillin-resistant *Staphylococcus aureus* (MRSA) is responsible for skin and soft tissue infections with multi-resistance to many antibiotics. It is thus imperative to explore alternative antimicrobial treatments to ensure future treatment options. Nisin (NIS), an antibacterial peptide produced by *Lactococcus lactis*, was selected to combine with Oxacillin (OX), to evaluate the antimicrobial effect and potential mechanism against MRSA. The synergistic antimicrobial effect of OX and NIS was verified by Minimal Inhibitory Concentration (MIC) assays, checkerboard analysis, time-kill curve, biofilm producing ability, and mice skin infection model in vivo. For the potential synergistic antimicrobial mechanism, the microstructure and integrity change of MRSA cells were determined by Scanning and Transmission Electron Microscope (SEM and TEM), intracellular alkaline phosphatase activity and propidium iodide staining were assayed; And transcription of *mecA*, main gene of MRSA resistant to OX, were detected by qRT-PCR. The results showed NIS could restore the sensitivity of MRSA to OX and inhibit biofilm production; OX + NIS can make MRSA cell deform; NIS may recover OX sensitivity by inhibiting the transcription of *mecA*. In vivo, mice skin infection models indicate that OX + NIS can substantially alleviate MRSA infections. As a safe commercially available biological compound, NIS and the combination of antibiotics are worth developing as new anti-MRSA biomaterials.

## 1. Introduction

*Staphylococcus aureus* (*S. aureus*), a major human pathogen, is capable of causing a range of diseases, such as skin and soft tissue infections, bacteremia and infective endocarditis [1]. Methicillin, the first semisynthetic β-lactamase-resistant penicillin, was designed for the treatment of β-lactamase-producing *Staphylococci* and used clinically in 1959. However, methicillin-resistant *Staphylococcus aureus* (MRSA) emerged two years later. MRSA strains are most often found as hospital- and community-acquired infections. According to China Antimicrobial Resistance Surveillance System, there had a 29.4% MRSA detection rate in 2020. The frequently detected MRSA clinically often make it responsible for many toxin-mediated diseases due to a repertoire of virulence factors and toxins, including toxic shock syndrome (TSS), staphylococcal foodborne diseases (SFD), and scalded skin syndrome, which may be a potential threat to public health.

The drug resistance mechanisms of *S. aureus* mainly include the production of antibiotic inactivating enzymes [2,3], efflux pumps [4], intracellular receptors with reduced affinity for antibiotics [5], and biofilms [6]. MRSA acquired resistance via *mecA* gene, and *mecA* encodes an alternative penicillin-binding protein (PBP2a) with lower affinity for most semisynthetic penicillin [7]. Biofilms are microbial communities that adhere to environmental surfaces, and the cells within biofilm are encased in self-produced extracellular matrix and significantly less susceptible to antimicrobial agents [8]. *S. aureus* could produce biofilms on surfaces of different medical devices, such as surgical instruments and bionic implants. If these biofilms are not effectively inhibited or removed, they may cause severe nosocomial infections in patients [9].

Nisin (NIS), an efficient and safe antibacterial peptide produced by *Lactococcus lactis*, has a wide spectrum of antibacterial activity against Gram-positive bacteria, and used as food preservative already. For antibacterial mechanism, NIS is similar to glycopeptide antibiotics, showing a strong affinity for lipid II (precursor of peptidoglycan synthesis); it binds to lipid II to form a complex, thereby hindering the normal synthesis of peptidoglycan, thus inhibiting the formation of bacterial cell walls [10,11]. NIS can also form transmembrane pores which dissipate proton-motive force and affect some energy-dependent reactions in cell, causing the release of cytoplasmic components and directly leads to bacterial death [12].

Presently, most antibiotics are ineffective against MRSA, while non-first-line antibiotics such as vancomycin, linezolid and daptomycin have strict application conditions and high incidence of adverse drug reaction, which lead to challenges in MRSA infection treatment. According to Clinical and Laboratory Standards Institute (CLSI), MRSA was defined as oxacillin (methicillin)-resistant or cefoxitin-resistant *Staphylococcus aureus*. Thus, susceptibility or resistance to a wide array of β-lactam antimicrobial agents may be deduced from testing only penicillin and either cefoxitin or oxacillin. The prevalence of drug-resistant bacteria has now globally become a well-known risk for infection control due to the shortage of available antibiotics and difficulties in new drug development. Therefore, drug repurposing and combination screens has become a promising substitute. It has been reported that the combination of NIS and some antibiotics had the synergistic antibacterial effect, restored the sensitivity of some antibiotics [13,14], but there have no comprehensively evaluated synergistic antibacterial effect of NIS with OX. We previously found the synergistic antibacterial effect of meropenem with NIS on MRSA [15]. As antibiotics, OX has a similar antibacterial mechanism with meropenem.

To evaluate the activity and explore the potential mechanism of OX in combination with NIS against MRSA isolates, the in vitro and in vivo assays were carried out. And our research can provide a data basis for the study of new antibacterial material and drug repurposing in MRSA control.

## 2. Results

### 2.1. The Synergy Antimicrobial Effect of OX + NIS against MRSA

The experiment grouping and abbreviations are BC (Blank control), PC (positive control), SC (solvent control), NIS (treated only by Nisin), OX (treated only by Oxacillin), OX + NIS (treated by Oxacillin and Nisin).

#### 2.1.1. Antimicrobial Effect against MRSA In Vitro

**Figure 1:**
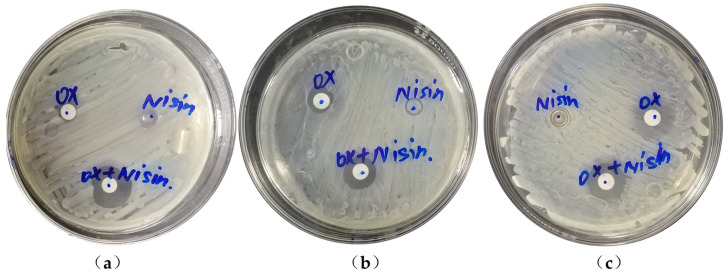
The change of inhibition zone of MRSA treated with OX, NIS and OX + NIS. (**a**) Yn2020043; (**b**) Yn2020051; (**c**) Yn2020070.

Base on the preliminary results of 16 MRSA strains using the disk diffusion assay (Kirby Bauer method) in Table S1. Compared with OX, the diameter of inhibition zone of the 16 tested MRSA strains were significantly increased (1.06 to 2.25 folds) in OX + NIS. And strain Yn2020043, Yn2020051 and Yn2020070, which are more sensitive to the OX + NIS treatment, were selected as representative strains for further evaluation (Figure 1).

#### 2.1.2. MIC and FICI of OX + NIS against MRSA

As shown in Table 1, the Minimal Inhibitory Concentration (MIC) value of OX against MRSA strains Yn2020043, Yn2020051, Yn2020070 and MSSA ATCC25923 are 32, 64, 16 and 8 µg/mL, respectively; for NIS, the MIC are all 12,800 µg/mL. While in the OX + NIS treatment, the MIC of OX against four strains decreased almost 4 times, with value of 8, 16, 4 and 4 µg/mL, respectively; and the MIC of NIS also decreased into 3200, 3200, 3200 and 6400 µg/mL, respectively. According to the results of fractional inhibitory concentration index (FICI), it is considered that OX + NIS has obvious synergistic inhibitory effect on the three MRSA strains, while additive effect on MSSA ATCC25923.

#### 2.1.3. Effect on Growth Curve of MRSA Treated with OX + NIS

**Figure 2:**
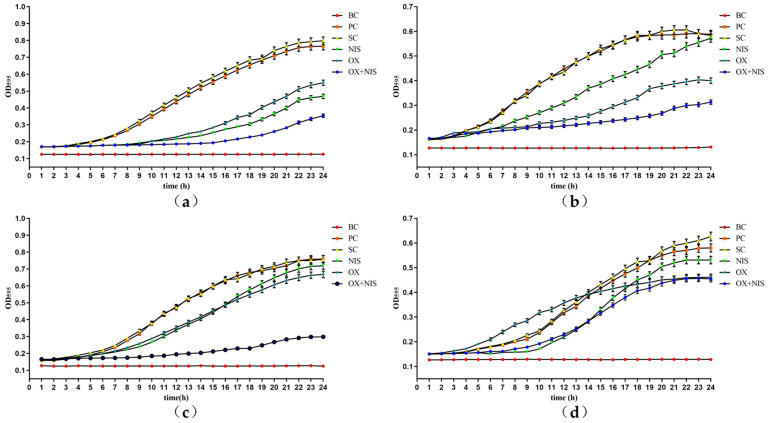
The growth curve of MRSA treated with OX, NIS and OX + NIS, with control of BC (Blank control), PC (positive control) and SC (solvent control). (**a**) Yn2020043; (**b**) Yn2020051; (**c**) Yn2020070; (**d**) ATCC25923.

The growth curves of MRSA treated with OX + NIS were shown in Figure 2, and the grow of three MRSA strains were all inhibited with the delayed logarithmic phase and stationary phase under OX + NIS treatment. In contrast with OX, NIS single treatment, the synergy antimicrobial effect of OX + NIS against MRSA Yn2020070 was the most effective, the cell growth and proliferation was severely inhibited.

#### 2.1.4. Effect on Biofilm Producing of MRSA Treated with OX + NIS

According to the classification criteria of biofilm producing ability, 3 strains of MRSA and ATCC25923 were all biofilm-positive strains with strong adhesion (Table S2). And 3 MRSA strains had higher biofilm producing ability than that of ATCC25923 (*p* < 0.001), where Yn2020070 was the highest (OD_595_ = 0.7704). 

**Figure 3:**
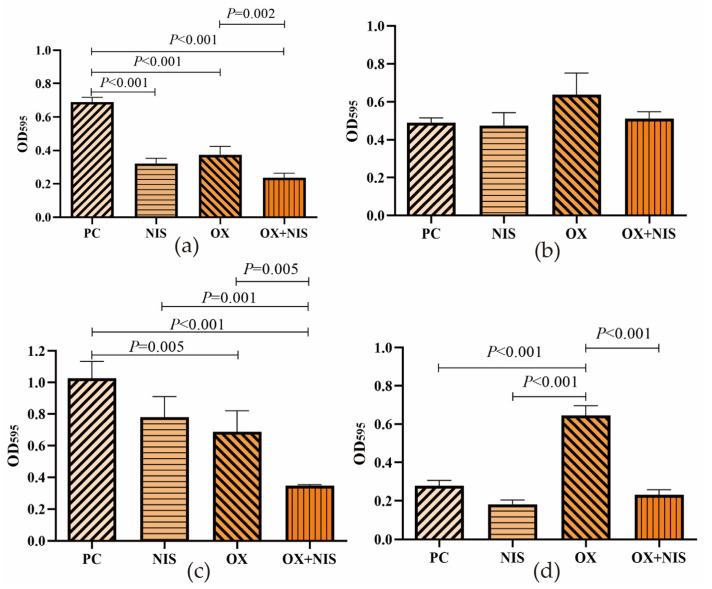
Biofilm producing of MRSA treated with OX, NIS and OX + NIS, with control of PC (positive control). (**a**) Yn2020043; (**b**) Yn2020051; (**c**) Yn2020070; (**d**) ATCC25923.

After treated with drug in 1/2MIC for 24 h, The biofilm producing in all treatment groups had decreased compared with PC (Figure 3). For MRSA Yn2020070, the biofilm formation in OX and OX +NIS group were statistically lower than the PC group with 35.59% and 66.07% decreasing (*t* = 3.417, *p* = 0.027; *t* = 10.398, *p* < 0.001); the biofilm formation in OX + NIS group was statistically lower than OX and NIS groups (*t* = 4.324, *p* = 0.012; *t* = 5.612, *p* = 0.005). Here we also found that the OX treatment had increased 131.94% of the biofilm producing in MSSA ATCC25923 compared with PC (*t* = 10.865, *p* < 0.001), while NIS and NIS + OX groups were all in decreasing of 34.46% and 17.19%.

From the bacterial growth curve and biofilm inhibition assay results, Yn2020070 showed the highest sensitivity to OX + NIS. Therefore, Yn2020070 was selected as a representative strain for further mechanism research.

### 2.2. The Synergy Antimicrobial Mechanism of OX + NIS against MRSA

#### 2.2.1. Morphological Changes of MRSA Cell

**Figure 4:**
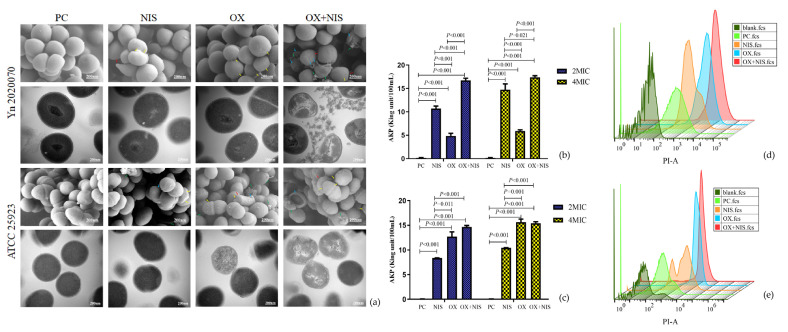
(**a**) Microscopic observation of MRSA Yn2020070 (line 1, 2) and MSSA ATCC25923 (line 3, 4) cells morphological changes treated with PC (column 1), NIS (column 2), OX (column 3) and OX + NIS (column 4) under SEM (line 1, 3) and TEM (line 2, 4). The small arrows indicated the cell damages; And AKP activity of MRSA Yn2020070 (**b**), ATCC25923 (**c**) under OX, NIS and OX + NIS; the cell membranes integrity of MRSA Yn2020070 (**d**), ATCC25923 (**e**) under OX, NIS and OX + NIS, was evaluated by the PI flow cytometric assay, showed in stagger offset histograms.

The cell damages under different treatment were observed with SEM and TEM (Figure 4a). MRSA Yn2020070 and MSSA ATCC25923 in PC cells were all in normal morphology with intact cell wall and membrane in spheroids, whereas strains exposed to OX and NIS singly were observed to have damaged cytoplasmic membranes and rougher surfaces compared with PC. Moreover, there had severe cell rupture in Yn2020070 exposure to OX + NIS, which may result in a serious leakage of cytoplasmic contents. For ATCC25923, it was more sensitive to OX than NIS, and showed more obvious damages in OX, NIS and OX + NIS than the corresponding treatment in Yn2020070.

It may suggest that the possible way of synergy antibacterial effect of OX + NIS may be related to the disruption of cell wall and increased the permeability of cell membrane, followed by disturbance of cellular integrity and the release of intracellular components, finally cause the death of cell.

#### 2.2.2. Integrity Changes of MRSA Cell Wall and Cell Membrane

The alkaline phosphatase (AKP) exists between the cell wall and cell membrane, and usually not secreted outside the cell. However, when the cell wall is damaged, AKP leaks and extracellular AKP activity could be detected in supernatant. As shown in Figure 4b,c, compared with PC, there had the increased AKP activity in all three treatments, where the OX + NIS group had the highest AKP activity indicating the more serious damages both under 2MIC (Yn2020070, 16.60 vs. 0.13, *t* = 17.088, *p* < 0.001; ATCC25923 14.65 vs. 0.04, *t* = 8.965, *p* < 0.001) and 4MIC (Yn2020070, 17.40 vs. 0.13, *t* = 21.095, *p* < 0.001; ATCC25923 16.39 vs. 0.04, *t* = 11.655, *p* < 0.001). For MRSA Yn2020070, the following damages was caused by NIS, then OX. For ATCC 25923, the following damages was caused by OX, then NIS. There had no statistical difference for AKP activities of OX + NIS group between 2MIC and 4MIC (*p* > 0.05), which may indicate that the disruption of cell wall integrity by OX + NIS was not drug concentration-dependent.

Propidium iodide (PI) is a fluorescent probe that can only penetrate cells with damaged cell membranes and bind to nucleic acids, so detecting changes in fluorescence intensity can reflect the integrity of cell membranes. As shown in Figure 4d,e, the percentages of PI-stained cells in NIS, OX, OX + NIS treated Yn2020070 were 19.0%, 58.2%, 81.7%, separately, while 16.8%, 51.2 %, 33.2% for ATCC25923. It is suggested that OX + NIS may damage the integrity of cell membrane.

#### 2.2.3. Transcription Change of *mecA* in MRSA

**Figure 5:**
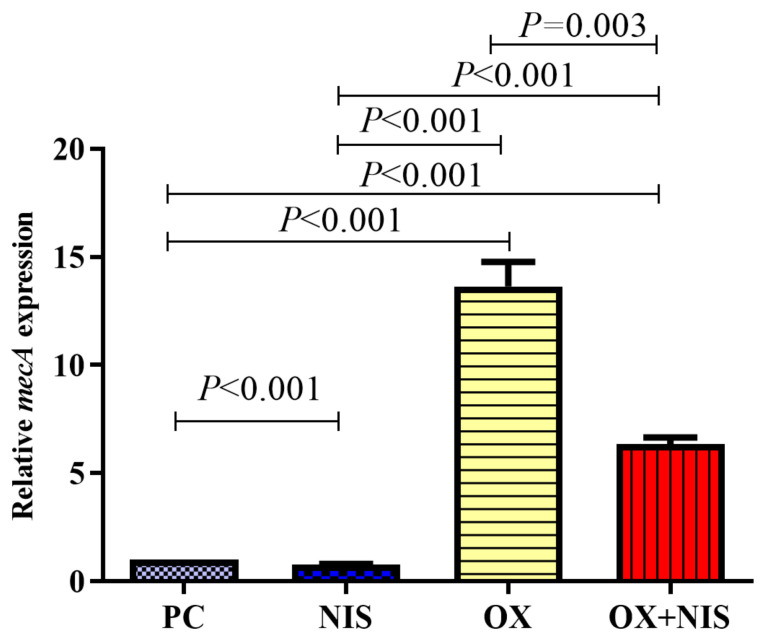
The *mecA* transcription in MRSA Yn2020070 treat with OX, NIS, and OX + NIS.

To explore the relationship of the sensitive of OX against MRSA combined with Nisin, the transcription of *mecA* gene in MRSA strain was quantified, and the qRT-PCR results are shown in Figure 5. Compared with PC, the fold change for the *mecA* transcription was significantly decreased in NIS group (1 vs. 0.77, *t* = 9.143, *p* < 0.001), while increased in OX group (1 vs. 13.65, *t* = 11.140, *p* < 0.001) and in OX + NIS group (1 vs. 6.36, *t* = 6.197, *p* < 0.001). According to the result, OX incubated with MRSA strain and induced the higher transcription of *mecA*, MRSA strain showed OX resistance; while when incubated with OX + NIS, the transcription of *mecA* decreased significantly (13.65 vs. 6.36, *p* = 0.003). It seems NIS could inhibit the transcription of *mecA* induced by OX.

### 2.3. Efficacy of OX + NIS in the Treatment of MRSA-Induced Wound Infections in Mice Skin

#### 2.3.1. Effect of OX + NIS Treatment on Wound Closure

**Figure 6:**
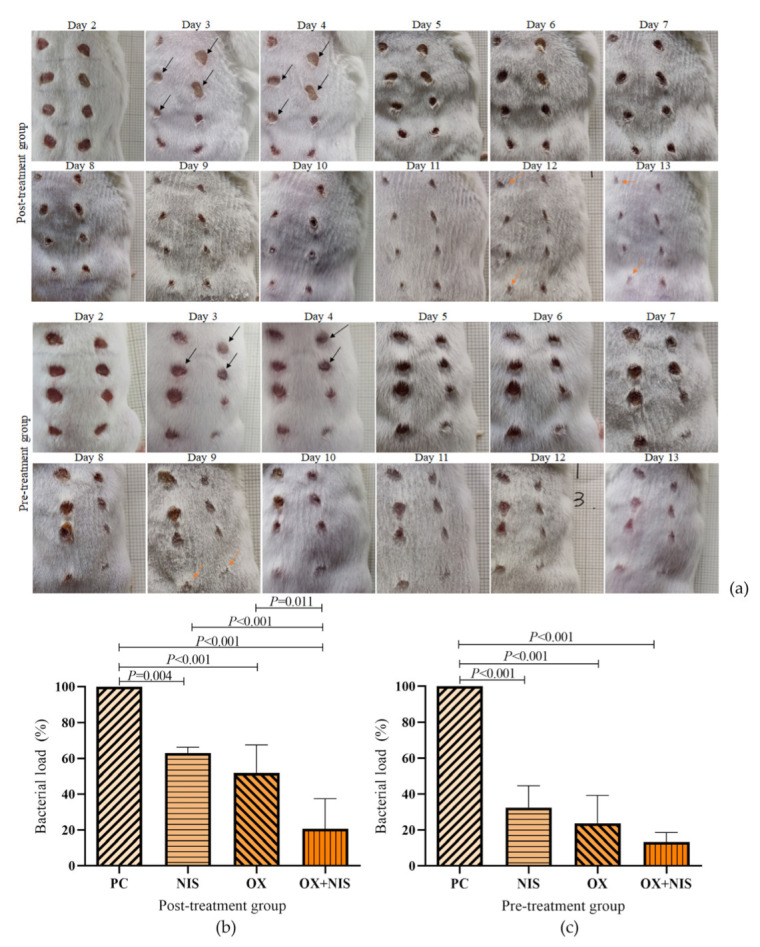
(**a**) Effects of post- and pre-treatment of OX and NIS alone and in combination on the healing of MRSA infected skin wound; At day 3, the bacterial load in MRSA infected skin wound were compared in post-treatment (**b**) and pre-treatment (**c**) of OX, NIS and OX + NIS.

Skin wounds on the back of mice were infected with 1 × 10^8^ CFU of MRSA strain Yn2020070. The progression of infection was evaluated daily for 12 days by visual observation and measurement of bacterial loading. Antimicrobial and control treatments were applied to the wounds 5 h after infection (post-treatment) or 5 h before infection (pre-treatment). As shown in Figure 6a, for post- and pre-treatment group, from day 3, OX + NIS significantly restricted the dermonecrosis and abscess area compared with PC, NIS, OX. On the day12 of post-treatment group, day6 of pre-treatment group, wound of OX + NIS almost healed, and the healing speed was same with BC group.

At day3, all treatments reduced the bacterial load both in post-treatment (Figure 6b) and pre-treatment (Figure 6c), as indicated by a reduced colony counts of Yn2020070 in the wound tissues. Compared with PC (100% survive), in pre-treatment wounds, MRSA strain had the survive rate of 23.61%, 32.54% and 13.36% in OX, NIS and OX + NIS; while in post-treatment, the survive rate was 62.97%, 51.91% and 20.67% respectively. Although the OX + NIS significantly inhibited the MRSA strain growth than PC, OX and NIS (*p* < 0.001) in pre-and post-treatment, the bacterial loads in the pre-treatment arelower than the corresponded post-treatment.

#### 2.3.2. Histopathological Change Analysis of OX + NIS Treatment on Wound Closure

**Figure 7:**
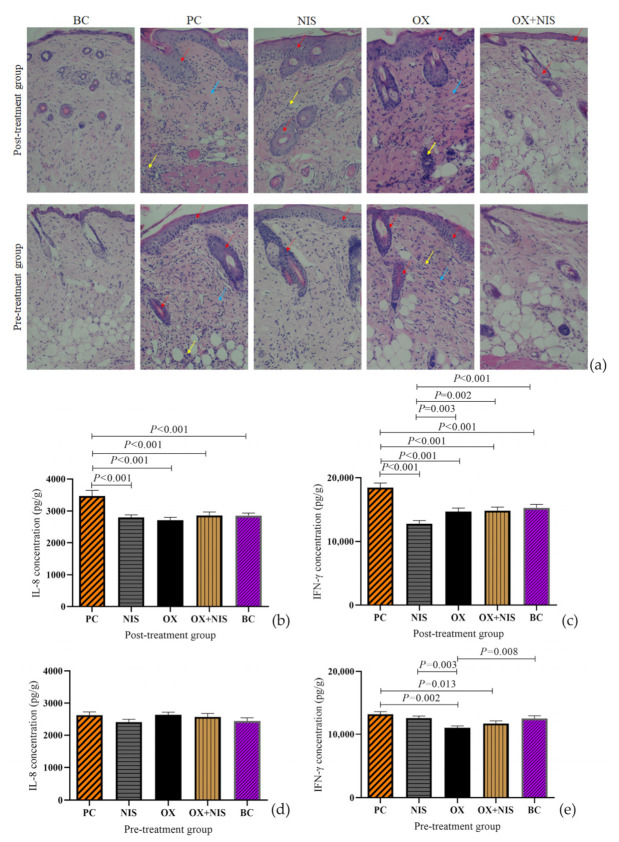
(**a**) Effects of post- and pretreatment of OX, NIS and OX + NIS on histopathological changes of MRSA infected skin wound (red arrow, cuticle; yellow arrow, inflammatory cell; blue arrow, histocyte); And the expression of IL-8 (**b**,**d**) and IFN-γ (**c**,**e**) in MRSA infected wound tissues in post-treatment (**b**,**c**) and pre-treatment (**d**,**e**) of OX, NIS and OX + NIS.

On day3, the treated wound tissues were collected and the Histopathological change were analyzed by Hematoxylin and eosin (H&E) staining (Figure 7a). The red, yellow and blue arrows represent the stratum corneum, inflammatory cell, and histiocyte, respectively. H&E staining revealed the degree of stratum corneum edema and inflammatory cell aggregation in the OX + NIS group were lower than those in other treatment groups, similar to BC group.

#### 2.3.3. Inflammation Cytokines Change Analysis of OX + NIS Treatment on Wound Closure

MRSA induced tissue inflammation had changed under different treatments, and the expression of inflammation cytokine (IL-8 and IFN-γ) were determined by ELISA (Figure 7b–e). In post-treatment, the expression IL-8 (Figure 7b) in PC group is significantly higher than OX, NIS, OX + NIS, BC groups (*t* = 6.678, *p* < 0.001; *t* = 6.057, *p* < 0.001; *t* = 5.136, *p* < 0.001; *t* = 5.512, *p* < 0.001); the expression of IFN-γ (Figure 7c) in NIS group was significantly lower than in OX, OX + NIS and BC group (*t* = 4.435, *p* < 0.01; *t* = 4.776, *p* < 0.01; *t* = 5.663, *p* < 0.001), there was no statistical difference between OX + NIS group and BC group (*p* > 0.05). In pre-treatment, IFN-γ (Figure 7e) of OX + NIS was significantly lower than PC group (*t* = 7.281, *p* < 0.05). In PC group, the expression levels of IL-8 and IFN-γ (Figure 7d,e) in the pre-treatment were only 75.59% and 71.41% of the post-treatment (Figure 7b,c), respectively.

## 3. Discussion

NIS has an inhibitory effect on Gram-positive bacteria including *S. aureus*, it can form permeable pores in cell membrane of target cells, and it can also inhibit the synthesis of bacterial cell walls, resulting in damage of cell wall integrity and intracellular components leakage, causing bacterial death [12]. Oxacillin is the semisynthetic β-lactam antibiotics, however, penicillin-binding protein PBP2a of MRSA has significantly lower affinity for β-lactams, allows cell-wall biosynthesis, which is the target of β-lactams. The results of in vitro experiments showed that NIS exhibited antibacterial activity when acting on MRSA and MSSA ATCC25923, which was consistent with the previous research reports [14,16]. The results of FICI assay showed that OX + NIS has synergistic inhibitory effect on the three MRSA strains. 

The 24 h growth curves showed that the combination exhibited synergistic effects at all stages, which may due to that OX and NIS were added at the early stage of bacterial proliferation, an important period for the formation of cell membranes and cell walls, and they are targeted by both NIS and OX. 

Biofilm formation is a significant challenge associated with treating device-related infections, and it is estimated that sessile bacteria in biofilms are almost 1000-fold more resistant to conventional antibiotics treatments and host immune responses than their planktonic counterparts [17]. Because the concentration of antibiotics decreases significantly when penetrating into the biofilm, bacteria are more likely to develop drug resistance when stimulated by low concentrations of antibiotics [18]. In addition, the metabolism of MRSA in biofilms is also reduced, hence the sensitivity to antibiotics is significantly reduced, which is the main reason for the difficulty in completely removing the foci of chronic infection [19]. Here, we demonstrated that, single OX treatment could inhibit the biofilm formation of MRSA Yn2020043 and Yn2020070, while significantly stimulated on MRSA Yn2020051 and MSSA ATCC25923, suggests that the responses of different *S. aureus* strains to OX may be quite different. In addition, OX + NIS could effectively inhibit the formation of biofilm compared with OX. Therefore, we speculated that NIS might play a major role in biofilm reduction, and mechanism needs to be further explored. Therefore, the combination of NIS with OX might represent a promising regime for the prevention of biofilm-associated infections. However, the microscopic observation of MRSA cells morphological changes through SEM and TEM, the intracellular AKP activity detection and membrane permeability assay all indicated that the target of OX + NIS most likely focused on the cell membrane and cell wall.

**Figure 8:**
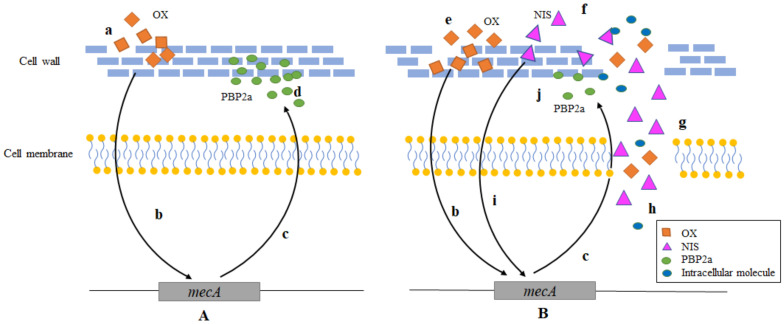
The schematic diagram of possible synergy antimicrobial mechanism of OX + NIS against MRSA. (**A**). The process of MRSA resistant to OX. OX contacts with MRSA cell wall to destroy cell wall synthesis (a); *mecA* is activated (b); MRSA expresses PBP2a (c), which has a lower affinity for OX; a large amount of PBP2a participates in cell wall synthesis and repairs the cell wall damage caused by OX (d); MRSA showed resistance to OX. (**B**). The synergy antimicrobial process of OX + NIS against MRSA. OX and NIS contact the MRSA cell wall, where OX destroys cell wall (e); NIS destroys cell wall and membrane (f), and forms pores (g); extracellular NIS and OX enter directly into the cell, caused leakage of intracellular molecules (h); NIS inhibits the transcription of *mecA* (i), reducing the synthesis of PBP2a (j), so the damage to the cell wall by OX cannot be adequately repaired. MRSA restores sensitivity to OX.

It is generally believed that PBP2a encoded by *mecA* gene, is the main reason for the resistance of MRSA to β-lactams [5], which is carried on a distinct mobile genetic element (SCCmec). And the expression is controlled through a proteolytic signal transduction pathway consisting anti-repressor (MecR1 and MecR2) and a repressor (MecI) [20]. qRT-PCR was used to quantify the transcription of *mecA* under OX, NIS and OX + NIS, and the transcription of *mecA* could be greatly induced by OX, while was significantly inhibited by OX + NIS (t = 6.197, *p* < 0.001). Therefore, we assumed that NIS could destroy the cell membrane and cell wall, then promote the antibacterial effect of antibiotics; NIS can reduce the transcription of *mecA* and restore the sensitivity of MRSA to OX. And the schematic diagram of the possible synergy antimicrobial mechanism of OX + NIS against MRSA was showed in Figure 8.

A mice skin infection model was used to verify the synergy antimicrobial effect of OX + NIS in vitro had similar effect in vivo. The results showed that OX + NIS could promote wound healing infected by MRSA. A study found that combination of NIS and some antimicrobial agents can significantly reduce the production of staphylococcal enterotoxin C of *S. aureus*, which may be related to the reduced healing time [21].

Furthermore, the efficacy of OX + NIS treatment of MRSA-induced wound infections in mice skin was determined, which including the therapeutic and preventive effects. The results showed that the wounds closure time of the OX + NIS group in both the post- and pre-treatment groups was faster than that of OX and NIS group, separately, indicating that the synergistic effect of OX + NIS still exists in vivo; And the efficacy of pre-treatment group was better than the post-treatment. Apart from individual differences, the mechanism may be that when OX + NIS is used in pretreatment, MRSA cell had to face the pressure of OX and NIS which have already penetrated into the wound skin tissue and cell interstices, and toxins are also diluted due to the formation of a protective layer by drug prevention. Therefore, bacteriostatic effect is achieved more quickly than post-treatment.

Under the post-treatment, IL-8, an important cytokine produced by leukocytes, endothelial or epithelial cells in response to pro-inflammatory stimuli that mediates neutrophil activation and transmigration to sites of infection, and the expression in PC group was significantly higher than that of other groups (*p* < 0.05). Therefore, the level of IL-8 can reflect the severity of the inflammatory response [22]. Some studies have found that the secretion of IL-8 increased under the stimulation of *S. aureus* enterotoxin C (SEC) [23], probably because the PC group may have the largest amount of SEC secretion. The expression of IFN-γ was the highest in PC and the lowest in NIS (*p* < 0.05). Studies have reported that NIS can reduce the secretion of IFN-γ. At present, IFN-γ is the information transfer station of cytokines and can regulate the secretion of many other cytokines downstream. But too much IFN-γ may lead to excessive immunity and damage to host cells [24]. Combined with the results of wound healing and H&E staining, it is concluded that the level of IFN-γ in OX + NIS group may be more appropriate for wound recovery than PC and NIS. Under pre-treatment, there was no significant difference in IL-8 of each group and IFN-γ between PC and BC.

This study found that the combination of OX and NIS can inhibit the transcription of *mecA* in MRSA, while further research is still needed on the specific regulatory pathways. Meanwhile, OX exhibited two distinct effects, inhibition and stimulation, on the formation/induction of *S. aureus* biofilms. However, the current research on *S. aureus* biofilm is not clear enough, and related mechanisms can be studied in future, such as the study of relationship between the phenotype of biofilms and related genotypes of different MRSA strains under OX stimulation. Hence, it is also necessary to conduct transcriptomics and metabolomics to explore the mechanism of OX combined with NIS on MRSA, which will be a focus of our future studies. At the same time, this study only used the mice skin infection model to explore the in vivo effect of OX + NIS, which has certain limitations. Therefore, other types of in vivo effect and mechanism studies should be arranged to more comprehensively evaluation. Based on the clinical significance of this study, nisin can be used for clinical treatment of MRSA, reversing MRSA to MSSA, and allowing the use of other beta-lactam drugs.

## 4. Materials and Methods

### 4.1. Bacterial Strains, Antimicrobial Agents, and Culture Conditions

Bacterial Strains: 16 MRSA strains were isolated from food specimens and identified in lab of Tongchuan City Center for Disease Control and Prevention, Shaanxi Province, China. Strain ATCC25923, a methicillin-sensitive *S. aureus* (MSSA) strain, was kept in our laboratory. Bacteria were grown in trypticase soy broth (TSB) overnight at 37 °C aerobically. Oxacillin (1 µg) was used in the disk diffusion assay (Kirby Bauer method). Nisin was dissolved in HCl (pH = 2), and filtered with a 0.22 μm filter to obtain 100 mg/mL NIS stock solution. 

### 4.2. Minimal Inhibitory Concentration (MIC) Assay

Base on the preliminary results of 16 MRSA strains using K-B paper diffusion method (Table S1). Three MRSA strains Yn2020043, Yn2020051, Yn2020070 were chose in the following experiments, and ATCC25923 was used as control.

MICs of OX and NIS were determined by the microdilution method according to the procedures outlined in the Clinical and Laboratory Standards Institute (CLSI). Briefly, bacteria cells were grown to log phase and resuspended to a cell density of 3 × 10^7^ CFU/mL (OD_600_ = 0.1) in TSB medium. Then, the serial dilution of OX and NIS, ranging from 0 to 0.256 mg/mL and 0 to 12.8 mg/mL respectively, was prepared in a final volume of 200 µL diluted in TSB medium. Each well contains 150 µL cell suspension (OD600 = 0.1) and 50 µL different concentration of NIS and OX, BC well contains 200 µL sterilized TSB medium, SC well contains 150 µL cell suspension (OD_600_ = 0.1) and 50 µL HCl (pH = 2). All plates were covered and incubated at 37 °C for 16–18 h. All MIC determinations were carried out in three duplicates.

### 4.3. Synergy Test of OX + NIS against MRSA 

The antimicrobial activity of OX + NIS was determined through a checkerboard assay as described [13]. Based on the MICs, 50 µL of different concentration of OX, NIS and OX + NIS with 2, 1, 1/2, 1/4, 1/8, 1/16 and 1/32MIC, separately, was added into the well with 150µL MRSA strain suspension (OD_600_ = 0.1) in a 96-well microplate; BC well contains 200 µL sterilized TSB medium; PC well contains 150 µL cell suspension (OD_600_ = 0.1) and 50 µL TSB medium. After incubating at 37 °C for 16–18 h, the FICI was calculated as follows [25]:

FICI = (MIC of Nisin in OX + NIS/MIC of Nisin) + (MIC Oxacillin in OX + NIS/MIC of Oxacillin).

The antibacterial effect of OX + NIS was defined as follows: (1) synergy (FICI ≤ 0.5); (2) cumulative effect (0.5 < FICI ≤ 1); (3) no interaction (1 < FICI < 4); (4) antagonism (FICI > 4).

### 4.4. Growth of MRSA Incubated with OX + NIS

The growth kinetics of OX, NIS and OX + NIS against MRSA strain Yn2020043, Yn2020051, Yn2020070 and MSSA ATCC25923 was determined as follows. Briefly, 150 µL of bacterial inoculum (OD_600_ = 0.1) was added. At MIC that shows synergy, 50 µL of OX, NIS and OX + NIS were added into the 96-well plate separately. And the BC, PC and SC were set correspondingly. Growth was monitored every 1 h at 37 °C for 24 h in OD_595_ with microplate reader (PolarStar Reader, Omega, Ortenberg, Germany). At least three independent replicates of each growth curve were obtained.

### 4.5. Biofilm Formation Assay

The biofilm formation of MRSA strain was assayed semi-quantitatively [26]. Glucose could increase the biofilm production and improve the sensitivity of experiment, so TSB medium containing 0.5% glucose was used [27]. A 150 µL of MRSA culture (OD_600_ = 1) and 50 µL of OX, NIS, and OX + NIS (1/2 × MIC of the synergy effect) were added in a 96-well plate. PC and BC were set, respectively. After incubation at 37 °C for 24 h, the content of each well was gently removed and washed three times with PBS to remove free floating planktonic bacteria, then dried. The wells were fixed with 200 μL methanol for 15 min and stained with 200 μL of 1% crystal violet for 15 min. The wells were washed three times and rinsed with 200 μL ethanol. Optical density of stained adherent cells was determined with an automatic microplate reader at 595 nm (Tecan Infinite M200, Männedorf, Switzerland).

The inhibition of biofilm by treatment (%) = (OD_595_ of PC-OD_595_ of treatment)/OD_595_ of treatment.

where treatment indicates OX, NIS or OX + NIS.

### 4.6. Microscopic Observation of MRSA Cells Morphological Changes Treated with OX + NIS

MRSA Yn2020070 and MSSA ATCC25923 were selected as representative strains for the study of inhibition mechanism. Bacterial cultures were exposed to NIS (3200 µg/mL), OX (8 µg/mL), a combination of NIS (3200 µg/mL) and OX (8 µg/mL), or PBS (control) for 10 h at 37 °C. Cells were collected by centrifugation at 8000 rpm for 15 min, then washed three times with PBS, fixed with 2% glutaraldehyde for 24 h at 4 °C. Microscopic observation of MRSA strain morphological changes under OX + NIS were examined by a Scanning Electron Microscopy (ZEISS, GeminiSEM 500, Oberkochen, Germany) and Transmission Electron Microscopy (TEM, Hitachi 7650, Tokyo, Japan).

### 4.7. Extracellular Alkaline Phosphatase Activity of MRSA Strain Treated with OX + NIS

An overnight culture of Yn2020070 and ATCC25923 were adjusted to OD_600_ = 10. Then OX, NIS, and OX + NIS (1/2 × MIC and 1 × MIC of the synergy effect) were added. After incubation at 37 °C for 2 h, bacterial supernatant (~0.5 mL) was collected for the measurement of extracellular AKPase activity using AKP kit assay (Nanjing Jiancheng Bioengineering Institute, Nanjing, China) [28]. The AKPase unit was defined as 1 mg of phenol produced by 100 mL of bacterial culture supernatant reacted with the substrate at 37 °C for 15 min. Strains treated with the same amount of sterile water were used as negative control. Each test was performed in three biological replicates.

### 4.8. Membrane Permeability of MRSA Strain Treated with OX + NIS

The integrity of cell membranes is evaluated by (PI flow cytometric assay [29]. PI is a fluorescent probe that can only penetrate cells with damaged cell membranes and bind to nucleic acids, so detecting changes in fluorescence intensity can reflect the integrity of cell membranes. MRSA Yn2020070 and MSSA ATCC25923 (OD_600_ = 0.01) were exposed to NIS, OX, OX + NIS (1.5 × MIC of the synergy effect), and the mixture was incubated at 37 °C for 4 h. PI staining was performed according to manufactures instructions (Sangon Biotech, Shanghai, China). The number of PI-stained cell was quantified by flow cytometry (BD Accuri C6 Plus, Franklin Lakes, NJ, USA).

### 4.9. Quantification of mecA Transcription of MRSA Strain Treated with OX + NIS

MRSA could produce PBP2a encoded by *mecA* gene, and the affinity of β-lactam antibiotics is reduced; while PBP2a can also catalyze the synthesis of cell wall, so MRSA shows resistance to Oxacillin. The transcription of *mecA* gene in MRSA strain treated with OX + NIS was quantified by qRT-PCR.

An overnight culture of Yn2020070 was incubated at 37 °C for 12 h and then adjusted to 1 × 10^6^ CFU/mL. Then NIS, OX, OX + NIS (1/2 × MIC of the synergy effect) were added, and incubated at 37 °C for 5 h. RNA extraction and quantification were performed according to manufactures instructions (Bacterial RNA Extraction Kit, Omega Bio-tek, Norcross, GA, USA; Reverse Transcription cDNA Kit; qRT-PCR Kit, Novo Protein Tech, Suzhou, China). Primers (Sangon Biotech, Shanghai, China) sequences used in this study were as follows: 16s RNA (5′-3′) F: GTG CCA GCM GCC GCG GTA A, R: GCG TGG ACT ACC AGG GTA TCT; *mecA* (5′-3′) F: GTA GAA ATG ACT GAA CGT AAG ATA A, R: CCA ATT CCA CAT TGT TTC GGT CTA A. 16s RNA was selected as the internal reference gene, and the relative mRNA expression levels of *mecA* were calculated according to 2^−ΔΔCT^ method. The untreated MRSA strain was used as control, and all tests were performed in triplicate [30].

### 4.10. Murine Skin Infection Model with MRSA

#### 4.10.1. Animal Used

Ethical clearance to conduct research on animals was granted by the ethics committee of Health Science Center, Xi’an Jiaotong University (2021-014). The 9–11 weeks SPF male Kunming mice (weighing 20 to 30 g) were purchased from Laboratory Animal Center of Xi’an Jiaotong University, and used for MRSA infection studies and housed in separate cages under controlled environmental conditions (12-h light/dark, 20–22 °C, 60–75% relative humidity). Every mouse per cage is housed during the whole experiment with free access of water and standard commercial mouse food.

#### 4.10.2. Skin Wound Generation and Infection with MRSA

Animals were acclimatized for 3 days before experiment. Before infection and treatment, mice were anesthetized with pentobarbital sodium by intraperitoneal injection (30 mg/kg body weight) and shaved on the back. Eight skin wounds were made on the back of each mouse with a sterile biopsy punch 4 mm in diameter, and each wound was inoculated with 6 μL of 2 × 10^8^ CFU/mL of MRSA Yn2020070. And the wound generation and infection procedure were referred as [31]. 

#### 4.10.3. Treatment and Evaluation of MRSA Wound Infections

To compare the therapeutic and preventive effects of OX + NIS, a post-treatment group (infected with MRSA firstly, 5 h later, treated with different drug) and a pre-treatment group (drug prevention firstly, 5 h later, infected with MRSA) were set. There had 8 wounds in the back of each mouse (Figure S1), and the wounds were treated with 12 µL PBS for BC, 6 µL PBS for PC; 6 µL of 6400 µg/mL nisin for NIS; 6 µL of 16 µg/mL oxacillin for OX; 3 µL of 32 µg/mL oxacillin + 3 µL of 12,800 µg/mL nisin for OX + NIS.

There was a total of 40 mice, 20 mice were in post-treatment group and 20 in pre-treatment group, 5 mice were used to observe the wound healing, 5 mice were used to detect the bacterial load, 5 were used for HE staining, and the other 5 were used for the detection of inflammation cytokines.

Wound healing observation: from days 2–13 after treated, the wound healing status were observed and photographed every day. 

Bacterial load detection: On the 3rd day, the wound skin tissues of 5 mice in each group were excised and separately immersed in PBS. MRSA colonies were counted of using MRSA rapid chromogenic medium (CHROMagar TA670, Paris, France).

Histopathological observation: On the 3rd day, the wound skin of 5 mouse in each group were excised and fixed in 4% paraformaldehyde. After H&E staining, the skin tissue slices were observed under inverted microscope (Nicon TS2, Yokohama, Japan).

Inflammation cytokines detection in the wound tissues: On the 3rd day, the wound skin of 5 mice in each group were excised and immersed in PBS separately, homogenized on dry-ice, centrifuged at 3000 rpm at 4 °C for 20 min. The concentration of IFN-γ and IL-8 in the supernatant were detected by ELISA kit (Shanghai Enzyme-linked Biotech, Shanghai, China).

### 4.11. Statistical Analysis

All experimental data are shown as means ± SEM, and statistically analyzed by SPSS 22.0 (IBM Inc., Chicago, IL, USA). The statistical significance was calculated by one-way ANOVA and differences were considered to be significant at *p* < 0.05. And GraphPad Prism 7 was applied for graphical plotting and analysis.

## 5. Conclusions

Nisin, is a peptide antibiotic that induces pore formation in bacterial membranes and inhibits peptidoglycan synthesis, was found to effectively and synergistically inhibit the growth of MRSA with combination of OX in vitro and in vivo. Moreover, Nisin can reduce the expression of *mecA* induced by OX and the biofilm formation, which may help circumvent the development of drug resistance and improve the antibacterial efficacy of conventional antibiotics. More importantly, Nisin combined with Oxacillin, also significantly alleviates MRSA infection in mice skin model. Hence, considering the relatively safety of Nisin, the combination therapy with Oxacillin is worth following up as alternative anti-MRSA infection regime, and it is expected to provide a research basis for the development of trauma medical dressings and materials for indwelling devices.

## Figures and Tables

**Table 1:** The MIC and FICI of OX + NIS against MRSA strains.

Strain	MIC (Alone)		MIC (Combination)		FICI	Inhibitory Effect
	OX (μg/mL)	NIS (μg/mL)	OX (μg/mL)	NIS (μg/mL)		
Yn2020043	32	12,800	8	3200	0.500	synergy
Yn2020051	64	12,800	16	3200	0.500	synergy
Yn2020070	16	12,800	4	1600	0.375	synergy
ATCC25923	8	12,800	4	6400	1.000	addition

## Data Availability

The data present in this study are available upon reasonable request from the corresponding author (hanbei@mail.xjtu.edu.cn).

## Supplementary Materials

The following supporting information can be downloaded at: https://www.mdpi.com/article/10.3390/ijms24076697/s1.
