# Supplementary material for: The Synergistic Antimicrobial Effect and Mechanism of Nisin and Oxacillin against Methicillin-Resistant Staphylococcus aureus
Source: Int J Mol Sci. 2023 Apr 3;24(7):6697. doi: 10.3390/ijms24076697 (PMC10094802; doi:10.3390/ijms24076697)
Supplement: Supplementary file 1 [file ijms-24-06697-s001.zip › ijms-2292408-supplementary.pdf]

Figure S 1. Model diagram of the therapeutic and preventive effects of NIS and OX alone and in combination on MRSA infection at murine skin wound site.

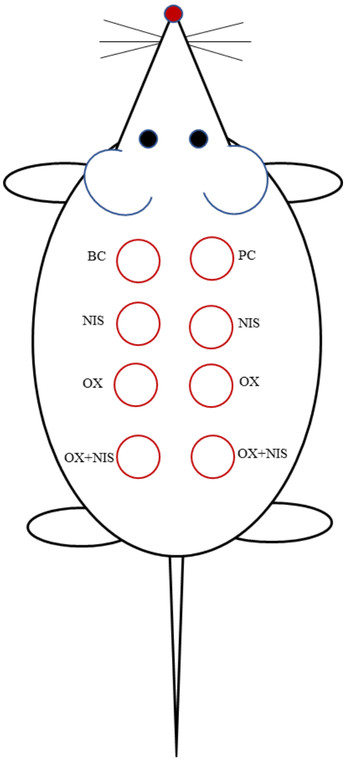

Table S1. Inhibition zone diameter increase multiple of OX+NIS group compared to the OX group of each strain

Bacterial strain	Diameter of inhibition zone (mm)			Increase multiple of inhibition zone
	OX	NIS	OX+NIS	
Yn2020050	12	8	14	↑*0.167
Yn2020043	8	8	18	↑1.250
Yn2020110	12	7	10	↓*0.167
Yn2020120	30	9	30	*—
Yn2020065	10	9	11	↑0.100
Yn2020067	22	8	22	—
Yn2020051	10	6	15	↑0.500
Yn2020070	10	6	16	↑0.600
Yn2020078	10	9	13	↑0.300
Yn2020004	9	7	13	↑0.444
Yn2020002	8	8	10	↑0.250
Yn2020098	18	9	19	↑0.056
Yn2020083	17	7	20	↑0.176
Yn2020130	15	7	20	↑0.333
Yn2020077	14	7	19	↑0.357
Yn2020093	19	7	19	—
ATCC 25923	16	9	18	↑0.125

Note*: "↑" means the increase of the inhibition zone diameter; "↓" means the decrease of the inhibition zone diameter; "-" means no change of the inhibition zone diameter.

Table S2. The biofilm formation ability of the strains

Bacterial strain	OD $_{sample}$ ($\overline{X}$±SD)	OD $_{critical\ point}$	biofilm formation ability
Yn 2020043	0.6006±0.123	0.0807	+++
Yn 2020051	0.4157±0.006		+++
Yn 2020070	0.7704±0.028		+++
ATCC 25923	0.3861±0.020		+++

Note*: The classification criteria of biofilm forming ability: The OD sample ≤ OD critical point was considered non-adherent, marked as (-); OD critical point < OD sample ≤2 OD critical point was considered weak adhesion, marked as (+); 2OD critical point < OD sample ≤ 4 OD critical point, considered moderate adhesion, marked as (++); OD sample >4 OD critical point, considered strong adhesion, marked as (+++).
